# Special issue on knowledge mobilization: Neonatal pain

**DOI:** 10.1002/pne2.12039

**Published:** 2020-09-03

**Authors:** Denise Harrison, Kathryn Birnie

**Affiliations:** ^1^ University of Melbourne Parkville VIC Australia; ^2^ University of Calgary Calgary Albert Canada

Incredible knowledge about neonatal and infant pain has been generated since the field's inception^1^; however, much of that research is not being put into practice or making it into the hands of people who need it the most, including healthcare providers, patients, and families.^2^ This means that while effective treatments exist for neonatal pain, suffering continues for premature, sick and healthy infants, and their families due to undertreated and preventable pain.^3^, ^4^

Knowledge mobilization is about making evidence "usable," by making knowledge accessible, understandable, meaningful, and useful for knowledge users.^5^ Knowledge mobilization bridges research, policies, and practices to improve outcomes in partnership with families, health professionals, researchers, educators, organizations, and policymakers, in this case, to ultimately improve pain for neonates, children, and their families.^3^

Solutions for Kids in Pain (SKIP; www.kidsinpain.ca) is a knowledge mobilization network whose mission is to improve children's pain management by mobilizing evidence‐based solutions through coordination and collaboration. SKIP is pleased to partner with pediatric and neonatal pain to introduce this first of two special issues focused on knowledge mobilization. These special issues highlight initiatives aimed to move knowledge in pediatric and neonatal pain into practice. This first special issue focuses on knowledge mobilization in neonatal pain (Part 1), with forthcoming issue focused on knowledge mobilization in pediatric pain (Part 2). Showcased is the incredibly diverse knowledge mobilization work being down in the field around the world. This includes efforts to engage and benefit varied knowledge user audiences, including health professionals, parents, children, and decision makers, addressing acute, procedural, and chronic pain in populations from infancy to later adolescence, and led by interdisciplinary teams.

The four papers in this special neonatal edition (Part 1) highlight how evidence about effective neonatal pain management can be mobilized via interprofessional interventions and social media.^6^, ^7^, ^8^, ^9^ Firstly, Balice‐Bourgois and her team report on the processes of developing a theoretically informed interprofessional intervention aimed at improving procedural pain management in a NICU in Switzerland.^6^ The content of the proposed intervention was then evaluated and approved by a panel of experts, healthcare providers, and parents. Secondly, Korki de Candido and her team report on an evaluation of the Portuguese version of the “Be Sweet to Babies” video in a postnatal setting in Brazil.^8^ The brief video, produced in 9 languages, is a knowledge translation tool, co‐produced with parents and targeted at parents, and demonstrates the use of breastfeeding, skin‐to‐skin contact and sucrose during painful procedures. In this case, the video was evaluated using a pragmatic pilot randomized controlled trial, on use of the pain management strategies during newborn screening in healthy newborns. In the third paper in this special issue, using the same video, Vieira and her team harnessed the power of social media, to evaluate the use of Facebook as a means of disseminating the video throughout the whole of Brazil, and to evaluate respondents’ prior knowledge, previous use of breastfeeding, skin‐to‐skin contact and sucrose, and intent to use these strategies in the future.^7^ The team reports a very large number of views, and 930 completed surveys, highlighting the huge potential of conducting knowledge mobilization research using nontraditional research methods. Finally, Bueno, Stevens, and the large ImPaC (Implementation of Infant Pain Practice Change) team's paper reports on the usability, acceptability, and feasibility testing of an online resource, targeted at clinicians, in a NICU in Canada.^9^ This resource is now being used as the intervention in a nationwide cluster randomized trial including 18 NICUs in Canada.

These four papers report on diverse and innovative research methods and interventions, conducted in three different languages (Swiss Italian, Portuguese, and English) in different parts of the world, but with the same focus; that of moving knowledge about newborn pain treatment into practice, with the aim of improving outcomes for newborn infants and their families. We are excited about this first special knowledge mobilization issue, focusing on newborn pain and hope our readers share this excitement. We are also excited about Part 2 of our special Knowledge Mobilization issue, focusing on children and adolescents.

## References

[1] Anand KJS , Roue J , Rovnaghi CR , Marx W , Bornmann L . Historical roots of pain management in infants: A bibliometric analysis using reference publication year spectroscopy. Paediatr Neonatal Pain. 2020;2:22‐32. 10.1002/pne2.12035 PMC8975229 35548591

[2] Chambers CT . From evidence to influence: Dissemination and implementation of scientific knowledge for improved pain research and management. Pain. 2018;159:S56‐S64. 30113948 10.1097/j.pain.0000000000001327

[3] Cruz M , Fernandes A , Oliveira C . Epidemiology of painful procedures performed in neonates: A systematic review of observational studies. Eur J Pain. 2016;20(4):489‐498. 26223408 10.1002/ejp.757

[4] McNair C , Campbell Yeo M , Johnston C , Taddio A . Nonpharmacological management of pain during common needle puncture procedures in infants: Current research evidence and practical considerations. Clin Perinatol. 2013;40(3):493‐508. 23972753 10.1016/j.clp.2013.05.003

[5] Graham ID , Logan J , Harrison MB , et al. Lost in knowledge translation: time for a map? J Contin Educ Health Prof. 2006;26(1):13‐24. 16557505 10.1002/chp.47

[6] Balice‐Bourgois C , Newman CJ , Simonetti GD , Zumstein‐Shaha M . A complex interprofessional intervention to improve the management of painful procedures in neonates. Paediatr Neonatal Pain. 2020;2(3):63‐73. 10.1002/pne2.12012 PMC8975212 35547023

[7] Vieira ACG , Bueno M , Harrison D . “Be sweet to babies”: Use of Facebook as a method of knowledge dissemination and data collection in the reduction of neonatal pain. Paediatr Neonatal Pain. 2020;2(3):93‐100. 10.1002/pne2.12022 PMC8975201 35547020

[8] Korki de Candido L , Harrison D , de Ramallo Veríssimo M de LÓ , Bueno M . Effectiveness of a parent‐targeted video on neonatal pain management: Nonrandomized pragmatic trial. Paediatr Neonatal Pain. 2020;2(3):74‐81. 10.1002/pne2.12023 PMC8975228 35547022

[9] Bueno M , Stevens B , Rao M , Riahi S , Lanese A , Li S . Usability, acceptability, and feasibility of the implementation of infant pain practice change (ImPaC) resource. Paediatr Neonatal Pain. 2020;2(3):82‐92. 10.1002/pne2.12027 PMC8975234 35547024

